# Toward a sustained recovery of the lodging sector: a management path to lessen the Corona Variants upshots

**DOI:** 10.1186/s43093-022-00171-3

**Published:** 2023-01-05

**Authors:** Ola Abdel Moneim Emara, Hazem Tawfik Halim, Mohamed Samy El-Deeb, Yasser Tawfik Halim

**Affiliations:** 1 Faculty of Management Sciences, October University for Modern Sciences and Arts (MSA), Giza, Egypt; 2 Faculty of Business Administration, Economics and Political Science, The British University in Egypt (BUE), Cairo, Egypt

**Keywords:** Lodging sector, Sustainability, Corona Variants, good health and well-being, Managing emergencies, Pliability

## Abstract

The lodging industry is an important source of national income being a main ingredient for tourism, also, as it contributes to solving the problem of unemployment. Predominantly, the lodging sector supports the balance of payments. At this place, a reading to *“Alpha”; “Beta”; “Gamma”; “Delta”; and “Omicron”*—*Corona Variants* reflects significant implications. On the one hand, there exists a challenge to Egypt’s hotel industry following the pandemic attack; on the other hand, and by considering the case of relaxing the international restrictions, the international tourism is expected to recoup. Egypt has relatively benefited in the first half of 2021 when many Europeans escaped the lower temperature in winter in their homelands opting warm holiday destinations. The primary readings also expect the continuity in this increase. The macro-trend right here considers the shift in favor of a thoroughly managed risk with enhanced pliability, adopting a view of creating value, and increasing trust, while the micro-management considerations signify enhancing the sustainability of the sector performance by means of raising lodging hygienic attributes and approving innovative approaches. Luxury hotel, led by their executives, can significantly contribute and steer this trend mainly for cost tolerance estimates and for the potentiality of cost recovery. This study addresses the ways with which *“Alpha”; “Beta”; “Gamma”; “Delta”; and “Omicron”* Corona variants necessitate more sustained hotel management practices and helps to establish knowledge and techniques that assist the lodging sector in Egypt. A further goal of this study is to endorse the hotel managers’ impact on counteracting the upshots of Variants’ progressions. The study is multifaceted in “scale” and “scope,” since it is based on an investigation of the effects of large-scale *public health* emergencies in the lodging sector. A qualitative methodology is set via conducting online and offline surveys to examine how the pandemic affect lodging sector operations, and how far Egypt’s luxury hotel managers tolerate both burdens of managing the emergency and creating sustained plans for recovery. Henceforward, the study constructs a frame for sustained lodging sector recovery. The paper’s scientific value is that it functionalizes a broad inductive method of qualitative research in which a merge of the interpretation of management enabling factors and health enabling trends in the lodging sector and luxury hotel managers’ responses obtained through exploratory qualitative research is utilized to cluster the managers’ responses and thus generalize the information in the setting of a Framework for Health Crisis Management in the lodging sector. The Framework for Health Crisis Management also adds value as it amalgamates a combination of input factors of management and health enablers; a transformation via the change actions; an output of containing the effect of the pandemic through the enhanced managers’ capabilities and hotels’ health hygiene readiness; and an outcome implication of medium-term stabilizing effects that leads to an impact effect of sustained lodging sector recovery. This configures an input, transform (actions), output, outcome, and impact result chain. The study reaches the findings that sustainable recovery in the lodging sector—being sensitive to the uncertainty that the Virus “Variants” create—necessitates the merge between managing the emergency, adopting pliable approaches, alongside with the enhancement of the managers’ (hoteliers) role as a supporting premise.

## Introduction

*Corona “Variants”*: *“Alpha”; “Beta”; “Gamma”; “Delta”; and “Omicron”* gained ground at an exponential rate putting millions of people at risk around the world in a progressive and wave mounted manner. The tourism industry crippled because of the strict forced lockdowns and the enforced quarantines in many countries [88, 92]. To help ensuring the safety of tourists, many stringent precautions were implemented. Chi, et al. [17] and Huynh, et al. [48] stated that in accordance with legal requirements, all airline firms had to pound their taskforces, while airports, hotels, food and entertainment facilities had to close, and events and conferences had to be negated or suspended.

Extraordinarily modest lodging room occupancy rates—thirty percent or below—were noted [36, 68, 70]. The emergency left the industry with unknown future, and forced hotels to reappraise their abilities in order to continue existing [75]. As Corona Variants spread and travel restrictions increase, the global tourist and lodging business continues to face difficulties.

The global COVID-19 epidemic has not only posed a great threat to human health, but has also affected people’s production and lifestyles, necessitating an additional combination of the analysis of the supply and demand side on economic sectors [96].

Due to the Corona Virus epidemic, hotel occupancy rates have collapsed and events have been rescheduled or suspended. Revenue Per Available Room (RevPAR) at Marriott hotels decreased by nearly 90% in China and by 25% in the rest of Asia–pacific district [99], while leisure and business travel were put on hold owing to Corona concerns [95].

The consequences that were not anticipated by managers before the spread of COVID19 could be counted among the circumstances beyond anyone’s control. For instance, severe temperatures may raise mortality risk in low- and middle-income countries since they not only confirmed a correlation between the two, but also revealed varying impacts depending on the economic cluster to which the country belonged [78].

Since the virus spreads largely via human contact, the travel, tourism, and hospitality industries were among the most hit by the global economic crisis. Lessons are drawn to fortify tourism policies and procedures in the wake of the pandemic. As soon as the decision is made to rebrand, travel incentives should be introduced. New approaches that are needed to be innovative are also needed as it can be challenging to establish a favorable destination image after the emergence of COVID-19 [15].

The Covid-19 outbreak strikes hotels hard. Since its discovery in China, Covid-19 has caused over two million deaths. Viral storm disrupted worldwide economy, diplomatic networks, and lodging business. Covid-19 endangers flights, cruises, and hotels. Many motels have closed or reduced rooms. Without demand estimates, revenue management (RM) is evaluated. Hotels must be adaptable in implementing revenue-generating strategies to survive the pandemic. Pandemic slows the hotel industry, but RM is still needed. Many hotels have closed because to Covid-19. The lockdown canceled numerous events, including those for guests. Hotels are not as prepared for pandemics as other sectors. Hotel management needs a strategic resource for the epidemic. During the Covid-19 disturbance, numerous Egyptian hotels closed, causing job losses. The lockdown canceled conferences, meetings, and daily events. Due to its sensitivity and severe rivalry, the hotel industry has not received as much attention as other sectors when measuring efficiency, despite its outsized impact on the Egyptian economy [101].

As in general, epidemics, natural catastrophes, and terrorist attacks all posture a threat to the lodging sector [2, 15, 27, 30, 32, 71]. For that, as diverse sorts of catastrophes have varying effects on the lodging business, this requires hoteliers to act to handle the numerous issues posed by emergencies. Besides, the potential rebound effect of high-risk damaging factors must be prevented or slowed down, thus it is imperative that we learn from the experience and implement the necessary proactive measures [97].

As it is constantly perceived, Egypt’s hotel industry remains to be an important source of national income, being a main ingredient for tourism, also, as it contributes to solving the problem of unemployment. Predominantly, the lodging sector supports the balance of payments.

Approximately, 9.5% of Egypt’s labor force is employed in the tourist and hotel industry, which employs about 2.5 million people. With combined sales at chain and independent Egyptian restaurants reaching over US$ 4.6 billion in 2017, the hospitality industry is a significant contributor to Egypt’s GDP. However, because of the reliance on services, these companies are especially vulnerable to the effects of the COVID-19 pandemic. Especially considering the elevated business risk already present in the hospitality sector prior to the pandemic and its subsequent spread throughout the hospitality industry, according to reports from multiple analysts, Egypt’s tourism business is losing $1 billion USD every month due to the coronavirus pandemic [27, 32].

At this place, a reading to*: “Alpha”; “Beta”; “Gamma”; “Delta”; and “Omicron”—Corona Variants* reflects significant implications. On the one hand, there exists a challenge to Egypt’s hotel industry following the pandemic attack; on the other hand, and by considering the case of relaxing the international restrictions, the international tourism is expected to recoup. Egypt has relatively benefited in the first half of 2021 when many Europeans escaped the lower temperature in winter in their homelands opting warm holiday destinations. The primary readings also expect the continuity in this increase.

**Fig. 1:**
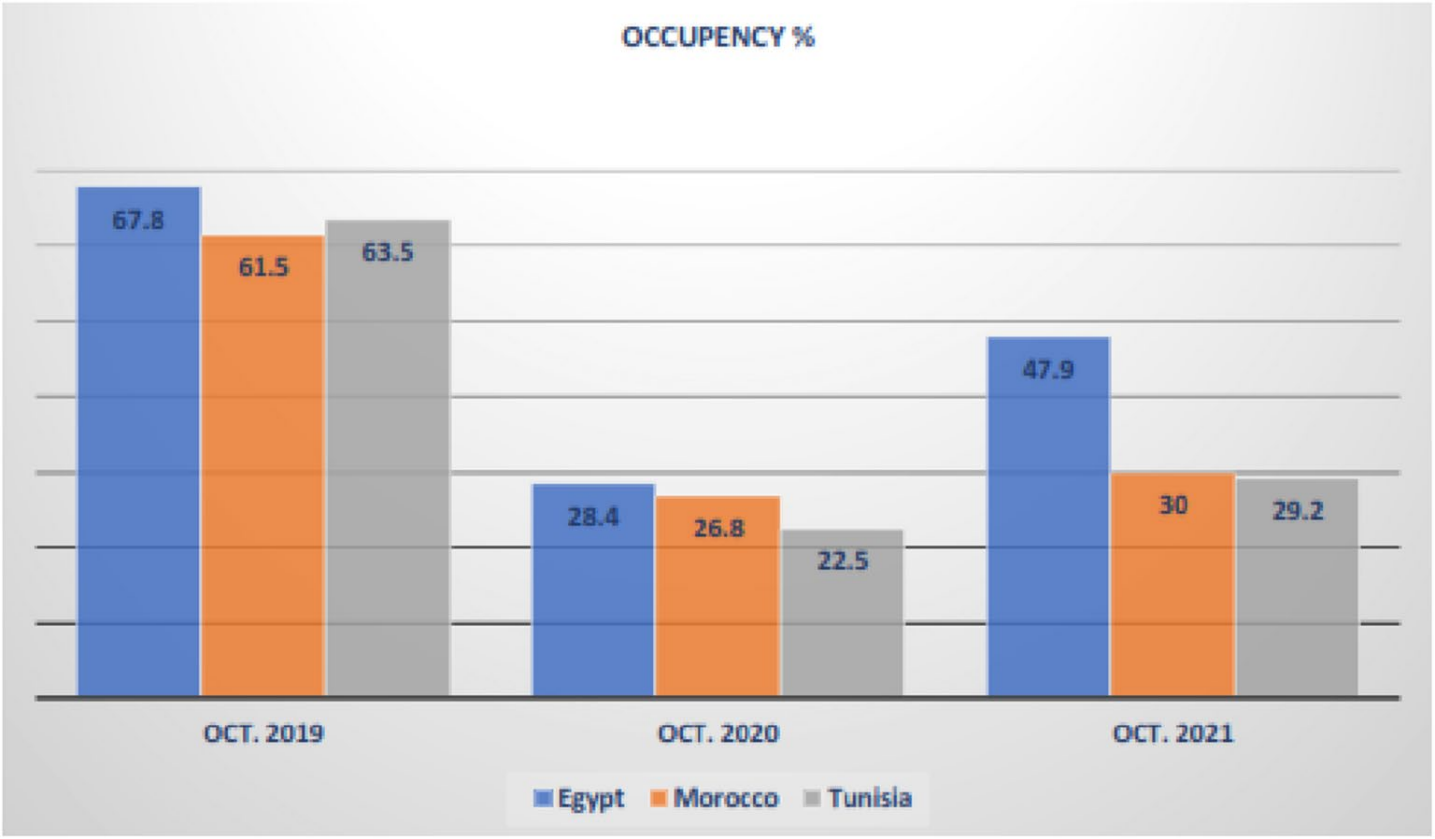
Comparative measures “North Africa Occupancy Rates for Lodging Industry”. Source: Egypt Leads North African Hotel Industry Recovery, STR, CoStar’s Hotel Analytics Firm 2021

In terms of the “Average Daily Rate ADR” for the period January to October 2021 was “1217.13” Egyptian Pounds compared to “1205.01” in 2020, while it remained lower than “1353.31” in 2019. Also, in terms of the “RevPAR,” the figures are “609.40” Egyptian Pounds, “342.04”, versus “917.53” for the same periods, respectively. In terms of “Occupancy %,” Egypt led “North Africa Countries” for each 10-month period January to October 2019 through 2021—Fig. 1 shows this comparison [106].

**Fig. 2:**
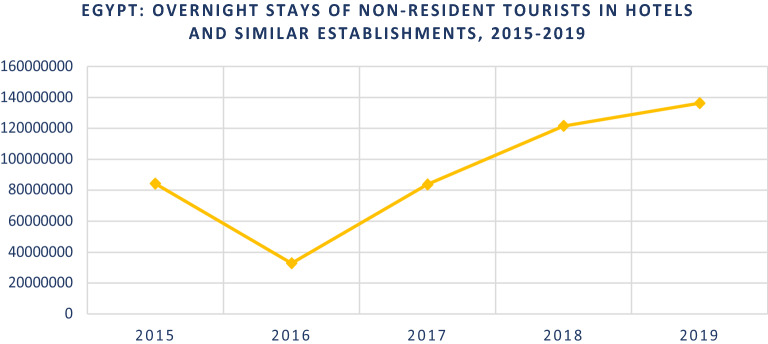
Egypt’s overnight stays of non-resident tourists. Source: UNWTO-Egypt-Country-Specific

The “World Tourism Organization UNWTO” (2019) referred to the 2019 increase in the overnight stays of non-resident tourists as shown in Fig. 2.

Prominently, the operational features of lodging raise the vulnerability of the tourism industry to the politico-economic, social volatility, natural disasters, along with pandemic Variants’ progressions. When such situations occur, tremendous drawbacks prevail. In terms of containing the drastic effects of emergencies on tourism destinations, the macro-level approach calls for key stakeholders to be prepared to deal with their distressing consequences, and make sure the industry’s survival does not suffer in the aftermath of these attacks [18]. In that the macro-trend right here considers the shift in favor of adopting an utterly managed risk with enriched pliability [37] and of embracing a view of creating value [28]. However, the micro-level approach primarily signifies thorough response methods to enhance the credibility of performance by means of raising health hygiene measures, and adopting technology innovative approaches [19, 75].

**Fig. 3:**
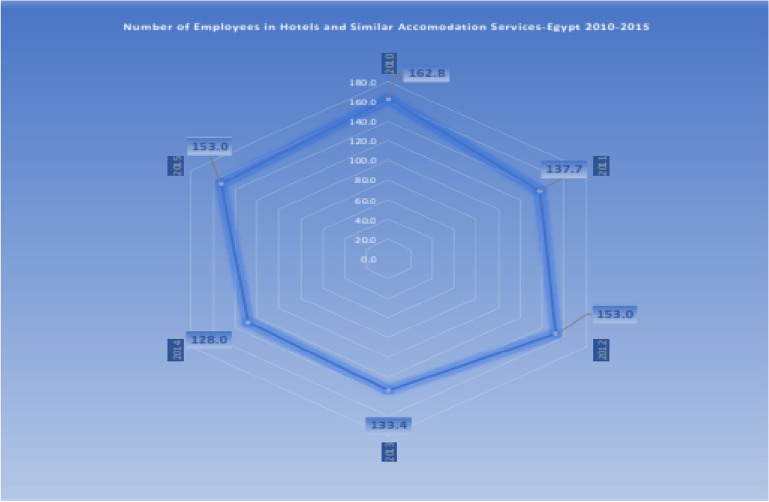
Egypt’s employees in hotels and similar accommodation services no. “in thousands”. Source: [104]

Concepts of *high public interest* continue to be prominent. In that the United Nations World Tourism Organization (UNWTO) calls for safeguarding “*workable-in effect-functions,*” while considering all beneficiaries and exchange parties. This covers *balanced job offering* and *retaining employees in the sector*. A wider scope of sustainable practice is necessarily bound to discussing revenue-generation offering within the frame of: *public awareness* and *participation* and *trust building*; *the observing of the consequences of management decisions*; *initiating proactive approaches*; as well as *adjusting measures when necessary*. Worth to mention also that while the *guest satisfaction* remains to be a significant target of the sector, the *guest orientation about sustainable relevant substances* is seen as crucial for maintaining the purpose of sector revival. UNWTO also sees *the utilization of new technologies;* the *enabling of competent human resources administration*; and *adopting innovative and entrepreneurial initiatives* as paths to sector renaissance [105] (Fig. 3).

As per UNWTO, “*Innovation and Sustainability as the New Normal*” have been called upon as “*priorities for holiday business retrieval*” (UNWTO, Global Guidelines to Restart Tourism, 2020). This draws to the operational features of lodging as a derivative of the tourism industry.

And hence, more pliable and sustained hotel management approaches need to be addressed to assist the lodging sector in Egypt. This can take place by fostering hotel managers’ contribution in counteracting the impacts of “*Alpha”; “Beta”; “Gamma”; “Delta”; and “Omicron”* Corona Variants.

As indicated by the World Health Organization (WHO), there are *public health management restrains* in the lodging sector that should be considered responding to the pandemic attack, this includes, yet, not limited to: allowing enough financial and manpower foundation as well as reviewing of the work flow and the tools and equipment; frequently revising and assessing the “measures in-use” to ensure low potentiality of the transmission of the virus; archiving the evidence and data that reflect the alternation of methods; adopting a multi-lateral communicative methodology within the entity; and paying attention to staff training of health and hygiene awareness [111].

Above and beyond, a prevailing case that exists in the lodging sector is that the cost structure is differently typified from other kinds of services by its high investments in long-lived assets versus the running cost of operations. Besides, there exists the condition that the personnel costs in the sector, being a labor-intensive sector, may reach the level of 80% of the total operating costs. All these put a cost burden, while the hope remains in the capacity of the hotels to generate revenues, even with small-scaled operations, as the food and beverage generated revenue are notably high [93]. Nevertheless, it is expected that the costs of operations will also augment in the near future due to the increased need for investing in new technology and in new health measures.

In the case of the lodging sector in Egypt, the study undertakes that luxury hotels, managed by their proficient executives, can significantly contribute and lead this trend—mainly for reasons of obtainable expertise, cost tolerance estimation, and the potentiality of cost recovery.

From this perspective, the study seeks the answers to the following investigative questions:

1. What are the management approaches needed to contain the lodging sector upshots *of the “Alpha”; “Beta”; “Gamma”; “Delta”; and “Omicron”* Corona Variants?
2. How did *Corona Variants* affect the lodging sector operations?
3. How far Egypt’s luxury hotel managers tolerate both burdens of handling the emergent circumstance and creating sustained plans for retrieval?

The study explores the effects of *large-scale public health emergencies* on the lodging industry responsive trends. The research develops a qualitative methodological tactic to examine how luxury hotel executives in Egypt can contribute to “*Alpha”; “Beta”; “Gamma”; “Delta”; and “Omicron” Corona* Variants’ response efforts. Managers’ experiences (MGR) are gathered and used to generate an interpretation of luxury hotel executives’ in-depth responses. A structured online and offline survey is conducted with three clusters of Egypt luxury hotel executives, covering three centers of hotel guest attraction in Cairo, Hurghada, and Sharm El Shaikh. The statistical readings produced are intended to deepen the arguments about managing emergency in Egypt’s hotel industry.

In turn, the study draws an analytical framework of the management enabling factors (*being handling the emergency, handling pliability, managers’ impact on managing emergency and pliability*); adjacent to health enabling trends in lodging industry (being service androids; lodging hygienic services; and improved standing of health), as drivers for managing change toward a sustained lodging sector recovery.

The study is formed of seven sections: It proceeds after the introduction to section two, which provides a reading of the management enabling factors. Section three reviews the health enabling trends in lodging industry. Section four elaborates the methodology, while section five signifies hoteliers’ reflections’ tracking. Section six proceeds to the study inferences by extracting an analytical framework for sustained lodging sector recovery. Finally, section seven provides concluding notes.

### Management enabling factors

#### Handling the emergent circumstance

An emergent circumstance that develops to a crisis, according to contemporary management literature, reflects a sudden disturbance that wrecks the foundations of a system and threatens its very existence [29, 51]. Also, framed as a significant occurrence that threatens organizations, corporations, or sectors, and is characterized by uncertainty about the reasons for the incident [19]. Past researches gave broad categorization of emergencies that develop to crisis instead of offering concrete emergency-handling models, yet, they seem to be intuitive, relying on previous experiences [19].

Yacoub and ElHajjar [100], Li et al. [57], Halim and Halim [39], Halim [42], and Halim and Emara [41] contended that in order to successfully handle an emergency, one must first look into the different response processes that are occurring. This line of thinking considered three stages: proactive planning ahead of an emergency, carrying out a strategic plan, and then measuring progress and making improvements [45]. Leta and Chan [56] described pre-emergency scheming as projections and estimates done ahead. Moreover, Sobaih et al. [87] disclosed the way to functionalize a plan for managing crises whether during, or post-crises. Finally, Chau, et al. [16] described a response and recovery stage in which an organization evaluates its efforts to reduce the negative consequences of an emergency.

Commonly, and with regard to lodging management studies, only few are dedicated to the micro-level, while usually studies examine how hotel managers can have an impact on the macro-level [26]. Nevertheless, emergency management is essentially dependent on managing people, this is where discrepancies between managers’ levels of commitment to learning and their actual application of learned methods tend to prevail. While a recommendation of the need for hotel managers to act in response to domestic and international threats such as communicable disease and terror incidents has been indicated by Donbak, et al. [25] and Mahmoud et al. [61], strategizing for management of the emergency is seen as crucial, as a tool for promoting hotels destinations to the international travel agents and vacation advisors.

For travel plans, digital marketing is becoming more popular as more people are using the internet. Many hotels have changed their business strategies as a consequence of the revolution in travel arrangements that has taken place in recent years. Online distribution channels have provided tourism firms with various benefits such as lower distribution costs, increased marketing opportunities, and more returns because of digital marketing, which has transformed how tourist and lodging services are marketed. Because the tourist and lodging industry is so diverse, and because there are so many different service providers, digital marketing is counted to be a valuable tool for both suppliers and customers to communicate information and do business. Because of this, lodging firms must better understand the needs and wants of their clients by creating and offering personalized items on their websites [44].

#### Handling pliability

A system’s ability to recover fully after disruption and reorganize itself such that it has mostly the same goals, structure, identity, and feedbacks is identified as pliability [19]. There is better guidance in Pablo and London [72] where the authors argue that maintaining pliability means managing change and implementing adaptive and creative solutions. Administrative pliability models consider the capability to manage vulnerabilities and dangers by preparing for them with long-term planning and the ability to alter course in response to changing conditions. Emergency management is connected with changes brought about by exceptional circumstances, while the phrase “unprecedented and incremental change” denotes pliability. A number of different measures for ensuring the safety of the system detected as learning, situation and context analysis, threat detection, proactive posture, planning methods, and recovery priority are all vital indicators of pliability [19]. Pablo and London [72] characterized pliability as being a scheme of dynamism, which outlines a hotel’s potential to analyze, develop, adjust, and deal with emergencies in a collaborative manner.

Many tourism-related reviews that consider pliability concentrate on sustainable dimensions and context perspectives of the event of political or economic crises, such as terrorism, and how the company responds [19, 65, 103]. Shrestha, et al. [85] claims that the tourism field has been neglecting the connections among emergency management and pliability. Tourism is strongly linked to a distinct set of unique qualities, and as a result, it has triggered the need to study pliability and evaluate the effectiveness of managerial growth and managerial preparedness in preparation for sudden crises.

Coping with the unexpected is one of the key components of pliability [63]. Workforce/employee/human resources practices encourage a workforce that has the capacity and promptness to adapt and support it with practices that are more optimal [8]. Knowing how to handle emergency situations is valuable for hotel managers, as it enables them to identify changes and minimize interruptions [12]. Pliability requires ongoing learning, adaptability, and responsiveness alongside adequate organizational resources such as training, as mentioned by Ashmond et al. [8]. Leta and Chan [56] and Lv et al. [60] stated that success in the hotel industry depends on proactive leadership that ensures both employees and the hotel are safe and provide excellent service. The presence of people who are well-informed, up-to-date, physically and mentally well, and talented, is a huge asset in times of emergencies.

#### Managers’ impact on handling emergencies and pliability

Ten managerial functions, ranging from interpersonal to informational and decisional, are mentioned by Dietzmann and Duan [24]. Following this by more elaboration in Smith, et al. [86], a methodology with three separate components: purpose, perspective, and position, is initiated, emphasizing that individuals, in their sociocultural setting, can be impacted by many occurrences, this can be altered by numerous emergencies [49].

Constant change is a critical component of the managerial role; yet there are no frameworks that comprehensively address both managerial emergency management and manager pliability [74]. Based on what Hahang et al. [38] stated, managers have several responsibilities and sets of talents that are influenced by their context and surroundings, with major categories being: operational, managerial, leadership, human, and IT abilities. Other studies stress the importance of managers having a strong work-life balance and the ability to use strategic and human resource management [83]. According to Heinze [47], it must be recognized that these roles are absolutely vital during an emergency, and that a manager needs to show ability to identify and implement both traditional and unconventional approaches to plans to achieve sustainable and operational transition.

To this end, successful management also requires managers to show, not only that they have a sophisticated and comprehensive talent management pervasiveness, but also that they have a multicultural perspective; that they can keep focusing on people; that they acquire knowledge of technology; and that they possess leadership abilities [20, 102].

Managers’ responsibilities in lodging research are among the most critical research interests [3]. An essential part of being a hotel manager is being able to expect, plan for, and provide training, to help avoid and reduce harm from various potential crises [87]. Salman et al. [81] found that managers’ responsibilities are considered as extremely complex undertakings that require judgments on a number of fronts that are mainly centered on policies for operations. Emergency management and pliability-building rely on managers being willing and determined to address the emergency and their ability to implement strategic skills across the board [72], while job security factors are suggested by Ashmond et al. [8] to make managers become more inclined to use constructive strategies.

Managers consider a variety of responsibilities in order to reduce the emergencies’ consequences, minimize negative effects, and steer the business through the phases after and before the emergency [56]. Preparing for crises is a critical responsibility of hoteliers [56]. Susilo and Prayudi [90] ascertain that seventy five percent of accommodation managers received emergency planning training, with comprehensive work out that highlight and address possible function inadequacies amid crises, consequently, hotel executives and staff members gained a lot of confidence in their emergency management experience. The use of shared responses and the dependence on social loops are emphasized as means of powerful uniting with outer schemes in the framework of the Corona Variants or any emergency progression [58].

A careful focus on the outer schemes will make it possible for hoteliers to estimate the unforeseen contextual changes and dangers, whereas the surface understanding of the contextual clues may raise the possibility of failure in realizing the organizational vulnerability to emergencies, with all the possible negative consequences [4]. According to Unterganschnigg [94], the denial of the possibilities for failure commonly lead to fragile systems, with both poor adaptation, and poor promptness to attacks.

Managers’ impact is significant, as the international experience reveals. With extremely high standards of service, the restaurant industry (RI) in India could be seen as a benchmark for significant management enabling success. RI in India is expected to continue rising in the future years. In India’s metropolises, restaurant culture has become more prevalent. The RI’s recent technological development has changed restaurant services, creating a big market for the RI in India. India’s popularity as a tourist destination has also contributed to RI’s rise. The RI has grown rapidly, considering the case that in the service industry, restaurants need to deliver outstanding service. Customers are pickier than ever when choosing a restaurant. As part of the hospitality sector, restaurant customers’ opinions on services and prices are crucial to customer satisfaction. Customer’s perceived value (CPV) quotients are important for measuring satisfaction because customers think they are getting a good deal for the services they pay for Ref. [10].

Unterganschnigg [94] pronounced three characteristic stimuli that describe a person’s behavior in emergency management, being, the evaluative thoughts and ideas, judgmental commons, and the exposure to similar circumstances. Inspirational and social binds, human communication modes; preparedness that rely on collective actions; are being emphasized as defense mechanisms [77, 84]. Managing pressure and the ability to reach well-adjusted solutions in tough times, the ability to communicate with subordinates on an emotional level, more and above the keep these sentiments adjusted toward optimistic approaches, all are seen to be necessities for hoteliers in times of devastations [43, 82, 94].

Managers must be able to show both mental and decision-making abilities in order to cope with pressure and calamities. Unterganschnigg [94] stated that the greater the organization’s unfamiliarity with the event, the greater the need for change and development managers to deal with the incident. Besides, the size and nature of organization can have an impact on emergency management and managerial impact [34, 94]. Huge organizations typically have a multi-divisional arrangement and collaboration in tactical synchronization, implying larger resiliency and/or managing catastrophe schemes and abilities. Ashmond et al. [8] argued that superior hotels (such as global chain-associated establishments) have advanced stages of directorial adaptability with a bigger opportunity to endure emergencies because they grasp additional investment, and are well organized. Smaller and medium enterprises businesses, conversely, may have additional simple business cooperation with catastrophe controlling as part of the manager’s role [22]. Managers are probable to accept a more HRM method in this situation, which adds to the member of staff motivation and rises administrative obligation in times of emergencies [8].

### Health enabling trends in lodging industry

#### Service androids

Succeeding the Corona Variants or any similar emergency progression, there would exist a large opportunity to develop digital and artificial intelligence (AI) marketing considerations, large data algorithms, and AI-driven cybernetics [7, 13, 66]. Since this virus is extremely infectious through person-to-person transmission, individuals have been advised to limit direct communication and surge social distancing [46].

The Variants’ progressions are probable to speed the implementation of digital and AI marketing, big data analytics, and cybernetics technology in the lodging sector. The evolving hotel service supports hoteliers with a chance to please and excite visitors in unique ways through the use of digital AI marketing technologies [5]. The application of digitalization, AI and androids in lodges is an inventive conception of service [59] and a rising research topic [62]. Numerous researches have inspected the usage of artificial intelligence and androids in lodges from a variety of marketing viewpoints. Along with Lukanova and Ilieva [59], invention processes involving androids can help hotels improve their long-standing effectiveness. Additionally, six features that determine the advancement of service androids in the lodging industry have been identified; these are support from the government, market growth; the growth of the cybernetics industry; lodges’ capacity for technological improvement; capital nurturing; and aptitude expansion [59]. Prentice et al. [76] evaluated this tendency from the perspective of the hotel staff where employees’ understanding of digital, AI and cybernetics strongly influences their intention to leave, while this may be restrained by features of organizational commitment; it can be negatively influenced by the competing psychological atmosphere. Carvalho and Sofia [14] identified future study criteria for the customer experience with interacting happening between a human and an android in relation to the influence of digital and multi-dimensional AI technologies on passengers’ experiences.

Hoteliers should continue to explore the applicability of mechanical artificial intelligence, thinking AI, and feeling AI in hotel service supply, service formation, and service engagement [66]. Several forms of artificial intelligence—mechanical, cognitive, and affective—may help to widen various scientific streams at the junction of humanitarian emergencies. Scholars might look into how mechanical AI can be applied to convert tedious social activities into non-contact frontline services. Mechanical AI can be applied in lodging sector to complete cleaning and disinfection activities or to aid hotel workers in carrying out their duties. Androids can help, for instance, to display the sanitation of high-density spots, monitor the quantity of dust or waste in corridors and lifts, in order to alert cleaning crews to places that need care. From such a standpoint, it would be beneficial to gather hotel customers’ ideas and sentiments about receiving an increasing number of non-contact services while having decreasing options to access services based on humans. Concerns are mostly centered on how artificial intelligence-driven cybernetics can effectively clean and sterilize lodge buildings, and also, how to establish suitable hotel facilities for artificial intelligence-driven cybernetics to operate in [1].

Due to the increase in the complexity of traveler demand after Corona, there should be a focus on declaring why artificial intelligence is crucial for hotel service customization, as well as by what means big data and analytical artificial intelligence marketing can be used to identify differences in individual preferences, for instance, when it comes to the criteria of hygiene and sanitation [66].

Hotel managers must strive to use big data to gain or preserve a competitive advantage over customized customer service [7]. Since information is the raw material on which digital and AI marketing is built, and since the use of thinking artificial intelligence marketing, expands in the lodging sector, the fields of business intelligence and big data-determined analysis must be improved in the lodging industry [7].

The drivers and obstacles to digital and AI marketing adoption in various categories of lodges and among explicit client segments are also intriguing. Due to the fact that digital and AI marketing applications can be regarded as either promoting or exacerbating service communications, the attainment of these inventions is deeply dependent on the distinct client relationship with technology [7]. Moreover, because the Corona epidemic and its Variants’ consequences have triggered worries, concerns; and depression among tourists, it is particularly essential to show compassion and respect in lodge services, and to delight clients throughout this difficult period [35]. As a result, a new area of investigation is to investigate what part “feeling AI” would play in hotel client care, and how “feeling AI” could upkeep human service employees in providing customized service and evolving customized relations with lodging guests.

Because of the benefits and drawbacks that digital and AI marketing might have on lodging service, attention should direct toward realizing whether the hotel economy benefits from service android development; or if it will be negatively impacted by it [59]. Hotels should also consider their preparedness in an “AI-based economy,” as well as their values surrounding the use of digital and AI marketing and AI-driven cybernetics in providing hotel services. A consideration should be given also to the potential impact of digital and AI marketing on a hotel’s dynamic capacities when confronted with a civic health emergency like Corona or any other emergency. A greater focus should be devoted to the indirect effects of digital and AI marketing in hotels on guests’ experiences, such as if the AI-human border conditions could provide increased guest value, and co-creation, in the wake of a public health emergency [50].

The digital health enabler has also added many innovative features. For instance, healthcare has been completely transformed by the introduction of electronic health record (EHR) systems. Healthcare is enhanced by these systems. The field of health IT is growing. As of 2015, 84% of non-federal hospitals used certified electronic health records (EHRs). There have been advancements in health IT. Health information technology (HIT) aims to reduce health inequities, promote population and public health, and improve EHR quality, safety, and effectiveness. Healthcare enablers were put into “meaningful use.” A lot of the new medical technology has been developed as a result of urgent healthcare problems. There are problems with privacy, interoperability, health information exchange in critical circumstances, and efficiency of health management, with possible aid from blockchain technology. Healthcare processes that are now inefficient may be enhanced by using blockchain technology. However, failure to successfully use blockchain technology can have severe consequences due to the magnitude of the required cultural, infrastructure, and business process shifts. Whether or not a project works on the blockchain depends on both internal and external factors, such as the skills of the designers and how easily blockchain laws can be changed [6].

It is worth noting that the last several decades have seen a dramatic shift in the way businesses connect with their customers as a direct result of the proliferation of digital technologies (DT) and virtual platforms. The trend toward embracing DTs like AI and robots is expected to continue as more convenient DTs like these become available. Yet it could be the case that deploying DT arbitrarily across different service settings could have a negative influence on customer happiness and loyalty. To reap the full benefits of implementing these DTs, service providers must also integrate them with their other human services. RI businesses have adopted mobile apps, touch-screen POS systems, and Wi-Fi hotspots in response to the DT. DT is becoming more valuable in this out-of-the-ordinary COVID-19 time when social isolation is common and contactless payment methods are mandated [10].

Nevertheless, digital and AI may gradually replace human labor, which may cause more workers to lose their jobs. Digital and AI creates a greater variability of consumer familiarities when serviced by AI cybernetics, instead of human service workers [5]. Differences in the outcomes of mechanization on hotel staff and guest service depends on the sort of artificial intelligence employed. For instance, the backstage procedures, such as apprising client files with data from the IT systems, form the preponderant part of the automation process. Furthermore, its implementation is uncomplicated, and the threat to hotel employees is low [13]. Perceptual observations can also be used to forecast customer behavior. Digital and AI assistants and customized recommendation systems can provide the opportunity to revolutionize the hotel industry, but their introduction needs to be managed slowly to avoid inefficient performance [13]. There would also be a need for focusing the cognitive insight, cognitive engagement, and process automation, on hotel management practices [60].

#### Lodging hygienic services

As declared by the World Health Organization, Corona Virus can be transferred by contacting virus-infected surfaces. Therefore, the importance of hotel sanitization management has amplified. Residues and microbiological contaminants accumulate on the hotel room’s surfaces make it more likely that hotel surfaces are unclean, contaminated, and serve as prospective disease vectors [9]. Additionally, through central air-conditioning, airborne transmissions could be another method of getting the Corona Virus [60].

Hotel guests are more inclined to pick comfort and hygienic services due to the prevalence of the Corona [89]. So, and as a result, hotels should undertake routine sanitary monitoring both by hand (with human staff) and with androids (via automation). The schemes that hotel cleaning personnel can utilize using AI-driven cybernetics should more explored. For instance, the use of sensors that assess the dust level in an area, and automatically operate in it, by working on the area or reporting to other cleaning teams. To increase their commitment to hygiene and safety standards, hoteliers must emphasize their loyalty to the requirements. A study published by Ahmad [2] indicated that hotels responded to the SARS and H1N1 flu epidemics in a similar manner- this is to assert the case that when it comes to communicable diseases, the presence of hygienic inspection in hotel facilities is positively correlated with reduced illness.

A hotel’s reputation strongly depends on the quality of the bedrooms and bathrooms [64]. General cleanliness and hygiene are significant in people’s choices of where to stay while traveling, as well as in regards to the customers’ contentment, feelings of satisfaction, and hence, loyalty [64]. Researchers have seldom, if ever, addressed the sanitation and cleanliness of specific spots in a lodge [9]. Surfaces that are in frequent contact with humans are more prone to become filthy through touch [87]. A surface may also serve as a platform for airborne viral propagation in unreachable places, such as the central air-conditioning system [60]. For that, incorporating an increased awareness of hotel hygiene in housekeeping procedures will enable hoteliers to develop more efficient cleaning and sanitation strategies for their properties.

The elevated request for hotel sanitation due to the Corona Variants’ progressions led to the need for enhanced hygiene and sanitation, with the main purpose of reducing the transmission of infections. From a marketing management perspective, the increased demand for hygiene and sanitation can be used as a marketing technique through, and after, the spread of a virus. Studies confirm this view by considering the materiality that younger visitors, and females of all ages, are prepared to spend extra money for better room sanitation [91]. Beyond just being clean, the conception of hygiene is concerned with the overall healthcare aspects. After the Corona Variants**’** progressions, hotel guests are anticipated to be increasingly concerned about finding healthcare services.

#### Improved standing of health

Throughout the Corona Variants’ progressions, many individuals begin to reconsider their lifestyles and take an active interest in their bodily and mental well-being [73]. Because this novel need emerges, serving customers by educating them on healthy living after the flu pandemic may be a succeeding trend in the lodging industry. One may expect an increase in the marketing mix of hotels’ meditation, digital detox, fitness, healthy food, and sleep hygiene programs. How hotels may tailor lines to enhance guests’ satisfaction and experience, how to better manage guests’ behavioral data and spending history record—such as the food bought, hotel fitness appointments, use of internal spa and wellness services; these analytics are expected to help creating personalized, health-based, facilities [31, 40].

Human beings have recognized the importance of nature and the environment as a result of the Corona Virus epidemic attack [54]. Subsequent the 2019 Corona epidemic, a rise in environment responsive types of service sector, such as eco-tourism and slow tourism is predictable, as these forms help promote ecological sustainability and human-nature harmony [98]. People’s environmental worries encourage hotels to adopt eco-friendly methods and procedures [80]. A future concern is centered on finding out why and how guests’ views on green hotel services vary across different types of hotels, and various travel activities, and hence hotels can build corresponding approaches [80].

Also, there is a necessity to create incentives for hoteliers and clients to act as a team in co-establishing the green value [35]. Furthermore, in a civic health catastrophe like the Corona Virus pandemic, the virus spreads between guests themselves, not only between guests and hotel staff [52]. As a result, there should be an emphasis on whether, and why, technology such as artificial intelligence, and AI-powered cybernetics, can ensure social distancing amid hotel visitors throughout pandemics, as well as on how lodges can apply effective physical distancing precautions between guests [21]

Medical hotels, which give healthcare services in a hotel atmosphere, have numerous qualities and benefits [53, 67]. Other hotel classes can benefit from the staff members that have superior healthcare understanding. In addition, there is a need to consider the possible role of hotels in emergency management. Biswas [11] framed the case that hotel and tourism business partnerships in coastal regions helped mitigate catastrophe risks, and that hotels can help mitigate emergencies by providing emergency shelters and short-term accommodations. This can create a strong mental image that links hotels with the promotion of health safeguarding.

## Methods

### Study design

A qualitative inductive methodology is employed. Following Thomas [107] and O’Kane et al. [69], the study was built on a broad inductive method where:

- The data examination was determined by both the research objective of how luxury hotel executives respond to “*Alpha”; “Beta”; “Gamma”; “Delta”; and “Omicron”—Corona Variants*, and the readings and the interpretations of the management enabling factors and health enabling trends in lodging industry;
- The development of categories of response from the raw data into a framework for sustained lodging sector recovery;
- Along this path, the raw data were coded by the researchers, and hence, the findings were extracted from interpreting the raw data.

The study questions were devised by adhering to “*literature review and analysis*” and “*preliminary discussions with experts in the field of lodging industry*”, also, based on “*authors previous experience with hotel managers, with a specific focus on critical operational areas related to the pandemic*” (i.e., people management, hygienic measures, social distancing).

The nature of the understanding gained from interviews depends on the study problem and the researcher’s discipline perspective. Some research is meant to test a priori ideas by using a highly organized interviewing approach with standardized stimuli (questions) and analysis. Other studies seek to comprehend a topic’s meaning and views. The research question and the researcher’s competence can both define the details. One form of study explores meaning and perceptions to better comprehend and/or develop hypotheses, while another evaluates an a priori hypothesis using a highly organized interviewing method with standard stimuli (questions) and analysis. Qualitative research starts with ideals. The quantitative approach uses statistics and is transferable. Qualitative research examines people’s thoughts, feelings, intentions, behaviors, and connections [55].

Also, as per Lê and Schmid [55] also indicate that the goal of exploratory research is to find answers to questions that have not been fully looked into before. Most of the time, qualitative methods are used in exploratory research. But a large sample size in an exploratory study may tend to reveal a quantitative sense. Researchers often use exploratory methods when they do not know how to solve a problem or when it is hard to get the right information. So, in order to do this kind of research, there is a need for either a broad idea or a specific subject to learn more about, but for which there is no existing information or paradigm. For the reasons listed above, this research followed a qualitative method.

Following Etikan et al. [33], a purposive sample (*expert sampling*) was used where all the research participants (experts of the lodging sector) were selected based on a profile that corresponds to the study objective, having the matching expertise and knowledge of the study topic due to the virtues the participants hold. Also, due to the lack of the current observational evidence, and the new perspective of the area of applied research.

### Data collection

The researchers used an offline and online survey to gather data about the phenomena. This involved running 101 offline and online questionnaires, with parley 85% online (86 respondents) and parley 15% offline (16 respondents), during which three groups of Egyptian Governorates were used as models of the lodging sector. The questionnaires began by place and occupation information; the common characterization of matters of experience; age group; alongside side with gender. More significant engagement was intended by requiring that the participant hoteliers elaborate on their experience with the emergency, and provide their reading of the potential progress of the lodging sector in Egypt.

There has been a comparatively limited amount of research conducted using empirical methods on participant experiences in qualitative research [23]. Yet, the study’s approach depending on the population and objective of the study, have been bases on a specified criterion of age, marital status, level of experience, employment status, and exposure to the period of managing the COVID-19 outbreak effects. In other words, because qualitative research results are based on close interactions with the participants, their experiences will be directly reflected in the results. As the data collection progressed, the authors noted saturation in the responses to come to an end of the investigative phase.

The process of collecting data took place between December 1,^1^ 2021 and February 28, 2022.

### Participants’ profiles

The coding process was made by identifying every respondent with a distinct code from MGR1 to MGR101. The great majority of respondents were male (93 percent). The majority of age group were between the ages of 50 and less than 60 years old (74 percent). In the survey, 76% of respondents said they had been Managers in luxury hotels for more than 10 years. All hotels (100%) are part of a multinational chain. Table 1 displays the clusters of interviewed hotels’ managers.

**Table 1:** Clusters of Interviewed International Hotel Chains’ Managers

Participant #	Cairo					Participant #	Hurghada				
	Years (Y) in Managerial Position				Age Interval and Gender		Years (Y) in Managerial Position				Age Interval and Gender
	Less than 5 Y	5–10	10–20	More than 20 Y	30 to > 40 40 to > 50 50 to > 60 Above 60 Male (M)/Female (F)		Less than 5 Y	5–10	10–20	More than 20 Y	30 to > 40 40 to > 50 50 to > 60 Above 60 Male (M)/Female (F)
MGR 1		X			40 to > 50 (M)	MGR 35			X		50 to > 60 (M)
MGR 2			X		50 to > 60 (M)	MGR 36			X		50 to > 60 (M)
MGR 3			X		50 to > 60 (M)	MGR 37			X		50 to > 60 (M)
MGR 4		X			40 to > 50 (M)	MGR 38			X		Above 60 (M)
MGR 5				X	Above 60 (M)	MGR 39			X		50 to > 60 (M)
MGR 6			X		50 to > 60 (M)	MGR 40	X				30 to > 40 (M)
MGR 7		X			50 to > 60 (M)	MGR 41		X			50 to > 60 (M)
MGR 8				X	Above 60 (M)	MGR 42			X		50 to > 60 (M)
MGR 9			X		50 to > 60 (M)	MGR 43		X			40 to > 50 (M)
MGR 10		X			40 to > 50 (M)	MGR 44			X		50 to > 60 (M)
MGR 11			X		50 to > 60 (M)	MGR 45				X	50 to > 60 (M)
MGR 12				X	Above 60 (M)	MGR 46			X		50 to > 60 (M)
MGR 13			X		50 to > 60 (M)	MGR 47		X			40 to > 50 (M)
MGR 14			X		50 to > 60 (M)	MGR 48		X			50 to > 60 (M)
MGR 15			X		50 to > 60 (M)	MGR 49			X		50 to > 60 (M)
MGR 16			X		50 to > 60 (M)	MGR 50		X			40 to > 50 (M)
MGR 17		X			40 to > 50 (F)	MGR 51		X			40 to > 50 (M)
MGR 18		X			40 to > 50 (M)	MGR 52				X	Above 60 (M)
MGR 19		X			50 to > 60 (M)	MGR 53			X		50 to > 60 (M)
MGR 20		X			50 to > 60 (M)	MGR 54			X		50 to > 60 (M)
MGR 21	X				40 to > 50 (M)	MGR 55			X		50 to > 60 (M)
MGR 22		X			40 to > 50 (M)	MGR 56		X			50 to > 60 (M)
MGR 23				X	Above 60 (M)	MGR 57		X			40 to > 50 (M)
MGR 24			X		50 to > 60 (M)	MGR 58				X	50 to > 60 (M)
MGR 25			X		50 to > 60 (M)	MGR 59			X		50 to > 60 (F)
MGR 26			X		Above 60 (M)	MGR 60		X			40 to > 50 (M)
MGR 27			X		50 to > 60 (M)	MGR 61			X		50 to > 60 (M)
MGR 28		X			50 to > 60 (M)	MGR 62		X			40 to > 50 (M)
MGR 29				X	45 to 55 (M)	MGR 63				X	Above 60 (M)
MGR 30			X		50 to > 60 (M)	MGR 64			X		Above 60 (M)
MGR 31			X		50 to > 60 (M)	MGR 65			X		50 to > 60 (M)
MGR 32		X			50 to > 60 (F)	MGR 66			X		50 to > 60 (M)
MGR 33			X		50 to > 60 (M)	MGR 67				X	50 to > 60 (M)
MGR 34			X		50 to > 60 (M)	MGR 68			X		50 to > 60 (M)

Participant #	Sharm El Shaikh				
	Years (Y) in Managerial Position				Age Interval and Gender
	Less than 5 Y	5 to 10	10 to 20	More than 20 Y	30 to > 40 40 to > 50 50 to > 60 Above 60 Male (M)/Female (F)
MGR 69		X			50 to > 60 (M)
MGR 70			X		50 to > 60 (M)
MGR 71			X		50 to > 60 (M)
MGR 72				X	50 to > 60 (M)
MGR 73		X			40 to > 50 (F)
MGR 74			X		Above 60 (M)
MGR 75		X			50 to > 60 (M)
MGR 76	X				30 to > 40 (M)
MGR 77			X		50 to > 60 (M)
MGR 78			X		50 to > 60 (M)
MGR 79				X	Above 60 (M)
MGR 80				X	Above 60 (M)
MGR 81		X			40 to > 50 (M)
MGR 82		X			40 to > 50 (M)
MGR 83			X		Above 60 (M)
MGR 84			X		50 to > 60 (F)
MGR 85				X	Above 60 (M)
MGR 86				X	50 to > 60 (M)
MGR 87			X		50 to > 60 (M)
MGR 88		X			40 to > 50 (M)
MGR 89			X		Above 60 (M)
MGR 90		X			40 to > 50 (F)
MGR 91		X			50 to > 60 (M)
MGR 92			X		Above 60 (M)
MGR 93		X			40 to > 50 (M)
MGR 94			X		50 to > 60 (M)
MGR 95				X	50 to > 60 (M)
MGR 96			X		Above 60 (M)
MGR 97			X		50 to > 60 (M)
MGR 98			X		50 to > 60 (F)
MGR 99	X				40 to > 50 (M)
MGR 100			X		50 to > 60 (M)
MGR 101			X		50 to > 60 (M)

### Data analysis

To identify the concepts that help understand managers’ impact, a four-step process was used. The questions were transcribed; initial ideas answered by managers were noted through repeated reading; coding the collected answers; then dividing these initial ideas into sub-themes based on manager thoughts, literature, and the study’s objectives.

Data analysis began with exploratory qualitative research to systematically interpret, then, generalize the information. There is some subjectivity involved in this procedure, and this can impact the validity and accuracy of the results. Nevertheless, in line with the research methodology, researchers re-checked the key themes and conducted an analysis of data and coding to verify the credibility of the findings.

## Results and discussion

### Hoteliers’ reflections’ tracking

#### Guests prospects and technology utilization

Hoteliers are expected to re-examine their client encompassing prospects and utilize advanced knowhow in order to improve the level of service. Table 2 displays the reflections of hoteliers grouped in six main clusters of thoughts.

**Table 2:** Reflections of Interviewed International Hotel Chains’ Managers

Clusters of thoughts	Supporting argument
Preparing to handle emergency and emergency controlling	“*Corporate offices established guidelines*” helped the greatest number of hoteliers who were previously employed by international chains to replicate a methodical path to managing the emergency. This went for elements related to the organizational functions and the hotel operative system. What helped was the previous involvement with emergencies accompanied with increased emergency promptness. Nevertheless, even existing plans were not always enough to deal with the size and scope of the emergency, that was, the enormity of the virus attach. The businesses responded to this earnestness by implementing novel procedures and standard operating procedures. In order to successfully implement contingency planning, guidance and support from corporate offices was extremely valuable. Additional processes and procedures were also updated on a routine basis by the heads of departments to manage emergency situations *Participants: MGR28; MGR34; MGT74; MGT85; MGR4; MGR25; MGR91; MGR3; MGR38; MGR71; MGR17; MGR63; MGR81; MGR52*
Pliable enablers	More than half of the managers exhibited strong levels of pliability and willingness to handle the consequences of a global pandemic. Managing people to return to business procedures, improving and maintaining administrative pliability, were the keys of strong managers. Managers functioned as change agents, as they had prior experience, knowledge, skills, and competencies to respond to an emergency. New operative tactics were regarded as opportunity to better market and innovate the customer service experience, and thus continue viable in a very challenging and unpredictable market. Managers with high level of pliability needed to be capable of going back to their preceding stage of overall performance, after going thru the responsive and rational mode of change; while additionally figuring out that administrative overall performance, engagement, and operations are all interrelated. Other than pre-planning, capacity-building was critical to help organizations adapt and change. Capacity-building helped organizations anticipate and handle changes as well as predicting their capability to recover from emergencies. In accordance with these ideologies, pliability was integrated into the managers' day-to-day routine to foster situational consciousness, boost adaptive capability, and detect conceivable susceptibilities. *Participants: MGR1; MGR3; MGR53; MGR14 and MGR46; MGR6; MGR66; MGR17; MGR72*
Steering day-to- day activities	*“Alpha”; “Beta”; “Gamma”; “Delta”; and “Omicron”—Corona Variants* had widespread effects on managers' activities, and on their daily practices, with certain abilities needed for pliability and approaching new structures and operative systems. The hotelier’s capacity to inspire and encourage groups in hard times, in addition to assure guests loyalty, relies heavily upon successful patterns of private interaction, and strong communicative norms. Organization had better demonstrate attentiveness and promptness by making changes across all levels, especially with regard to technology, human resources, marketing, and operations. Managers were aware of the current conditions and could notice danger signs, thus leading their business to be emergency-ready. Nevertheless, anxiety, tension, and difficulty planning ahead as a result of the unknown circumstance became a norm. Managers adopted a forward-looking stance, identifying the finest probable explanations for the industry and the staff by relying on their knowledge. Furthermore, a very important estimation to consider was that more hotels became more anticipated to implement Al technology and employ androids to deliver totally contactless services, including android assistants, face scan check-ins, android personal assistance, and other contactless offerings that will start to supplant human interaction facilities in the coming years *Participants: MGR100; MGR89; MGR55; MGR98; MGR61; MGR93; MGR57; MGR28; MGR75; MGR82; MGR14; MGR101; MGR41; MGR65; MGR90; MGR34; MGR80; MGR70; MGR58; MGR16; MGR34; MGR50; MGR26; MGR10; MGR60; MGR77; and MGR84; MGR3; MGR45*
Influencing central operating spheres	To ensure the financial stability of the company, managers should monitor four important functional areas: operations, marketing, and financial management. These areas were affected by the emergency. In light of the pandemic, a new business model is emerging. The business has to become more adaptable in the near future to cope with reduced demand. The issue in adhering to technology is to continue providing good service and social exchange with guests in spite of confronted complications. A core concept to hoteliers is to satisfy guests' needs and expectations. With the aim of eliminating the necessity for face-to-face contact whenever possible, it is expected that guests will not receive as much cross-communication with staff; guests will instead enjoy the personalized service of a concierge for the duration of their stay. While high ranked hotel chains dedicated departments that handle marketing activities; digital marketing algorism can help cater to different audiences. A price war between lodges is said to be inevitable irrespective of the path utilized to market hotel services. People management and staff levels were mentioned frequently as challenges alongside with efforts to keep labor costs under control. Yet; reducing layers of management will help reengineer reporting lines. Because of the present financial and economic stresses, the number of people who are unemployed in the luxurious lodging sector is probable to significantly increase, which means there will be a larger supply of available talent, candidates should also have the ability, willingness, and skill to handle complex work environments. *Participants: MGR86; MGR87; MGR1; MGR59; MGR97; MGR26; MGR1; MGR24; MGR99; MGR31; MGR34; MGR89; MGR3; MGR34; MGR68; MGR41; MGR78; MGR31; MGR69; MGR24; MGR79; MGR32; MGR39; MGR3; MGR50; MGR28; MGR32; MGR14; MGR13; MGR70*
Digital marketing and artificial intelligence (AI)	Hotel chains, marketing groups, and third-party reservation services are just a few of the methods hotels utilize to get their rooms into guests' hands. Hotels prefer working via travel agencies, tour operators, or incentive houses rather than directly. For their part, hotels are making use of the increasing number of electronic and digital distribution channels. In general, the channels are utilized for three things: segment the potential guest; informing potential guests about the hotel and speeding up the reservation process. In times of heavy competition or economic contraction, hotels may obtain a competitive edge by using digital and artificial intelligence marketing, as these channels serve as a realistic focal point for accommodations. When it comes to business, the travel and tourism industry was one of the first to take advantage of AI technology adoption as this information-rich industry relies on constant innovation in order to keep up with the ever-changing consumer demands while also providing a high level of customer satisfaction. Accessibility; public relations; relevance; transparency; partnership; convenience; intention to use web information; system quality; additional benefits; reservation times; are just some of the factors that must be considered in order for digital marketing to be successful and beneficial to hotels. *Participants: MGR2; MGR51; MGR101; MGR36; MGR62; MGR92; MGR39; MGR64; MGR96; MGR73; MGR88; MGR100; MGR54*
Estimate return for the sector	As service diversification in luxury hotel was addressed to match the shifting client needs, *luxury lodging was expected to return before other ranks of lodging, and will probably experience stabilized return within the medium term.* Besides, *hoteliers projected that a unique operational model is expected to emerge in Egypt's lodging industry.* With an expectation that Egypt's Lodging Sector recovery is likely to be recovering between 2023 and 2024, many hoteliers predicted that once the emergency is over, everything will return to normal. Others indicated that the lodging industry will be in survival mode depending on several factors. These factors include: the widespread of the rapid diagnostic test, the handling of the vaccination and the immunization of the virus, as well as national health policies. *Participants: MGR44; MGR6; MGR28; MGR14; MGR12; MGR19; MGR101; MGR50; MGR31; MGR32*

### Key findings

Innovation in research methodologies should always be front and center, as demonstrated by Lê and Schmid [55] and Reay et al. [79]. Thus, the emphasis should be placed on developing and employing research techniques that are novel to or infrequently employed within a given field of study. Qualitative research strategies necessitate novel use of methods and a creative mindset. Qualitative research techniques in the field of management study have the ability to be both flexible and innovative. And hence, this study attempted *improvement innovation in methods.*

This study offers a view of the luxury lodging industry at a time when most countries had just begun loosening travel restrictions, giving Egypt a snapshot of the market. The study considered the effect that emergency management and pliability strategies have on administrative consequences in the lodging industry. Additionally, given the complex nature of the Corona Variants’ attacks and their impact on the flow and nature of operations, the hotels’ planning process must be pliable and adaptable. A change may be indorsed via a result-chain repercussion. There are a number of separate but interconnected factors at play here, including management and health enablers as input factors; transformation via change actions; containment of the pandemic’s effect via improved managerial skills and hotel health hygiene readiness as output; and an outcome implication of medium-term stabilizing effects that lead to the impact effect of sustained lodging sector recovery.

The main outcomes of the study came to be in line with the study investigative questions, and are put in the frame of “*Alpha”; “Beta”; “Gamma”; “Delta”; and “Omicron” Corona* Variants repercussion on the lodging sector in Egypt. Hence, *sustainable recovery in the lodging sector—being sensitive to the uncertainty that the Variants’ progressions create- necessitates the merge between managing the emergency alongside with enriching pliability, while reinforcing the impact of hotel executives as supporting premise.*

The main areas of change management that have been realized:

A. *Managing emergencies by means of emergency planning* This is where the availability of generic emergency management and contingency plans (without Corona Variants specific—mainly due to earlier pandemics); along with hotel responses and actions by means of quick execution of directions, proper procedures, strong rules, introduction of new methods with hygienic policies, and the enforcement of new revised policies; *led to significant effects toward higher pliability; and hence, potentiality of sustainability of recovery of the lodging sector*.
B. *The impact of managers* Dedicated managers provided strong leadership in this emergency by remaining close by to workforce and making an earnest effort to boost their morale, managers acted as change agents, and remained in duty of formation and carrying out of achievement plan(s) in reaction to the emergency. On the positive side, managers developed new abilities in communicating with their subordinates; on the adverse side, pressure, nervousness, and aloneness, were among the consequences.
C. *Managing the hotels’ main activities* Food and beverage and housekeeping departments were most affected by the new instructions, and the stricter hygiene protocol. The new rules also had a bigger impact on the technology departments. Efforts for looking after staff retention and achievement of cross-departmental goals took place by implementing both salary cuts and flexible work schedules, functioning for the duration of the lockdown to retrain employees, developing cross-departmental capabilities and new standard for workplace hygiene, and hiring talented employees due to high unemployment. Price war and the “existence of the fittest” in sales materialized; managers’ detained accountable for the corporate existence; better use of online travel agencies and direct marketing and internet sales was adopted; sales efforts were related to info promotions aiming to return clients’ trust/persuading guests that lodging sector in Egypt is safe places to vacation without having a negative impact on their overall guest experience.
D. *Predictions for hotel recovery* the sector predicted to be recovered amid 2023 and 2024 up to 70% capacity, with reasonable hopefulness even with the damaging effect of Corona Variants to luxurious hotels; generally, managers trusted the sector is flexible to emergencies and skilled to get better rapidly.

### Study inferences

#### Framework for sustained lodging sector recovery

**Fig. 4:**
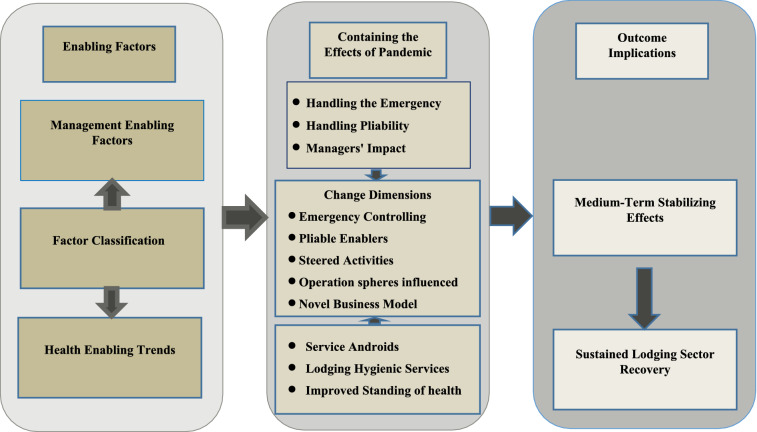
Framework for sustained lodging sector recovery. Source: authors' analysis

Combining the readings interpretation with the result of the qualitative research findings, the study resulted in sketching a framework for sustained lodging sector recovery. This is where management enabling factors (*being handling the emergency, handling pliability, managers’ impact on managing emergency and pliability*); alongside with health enabling trends (*being service androids; lodging hygienic services; and improved standing of health*) articulate new change dimensions of emergency controlling, pliable enablers, steered activities, operation spheres influenced, and novel business model. Hence, medium-term stabilizing effects materializes to allow for sustained lodging sector recovery (Fig. 4).

The change dimension, as the core of this framework, calls for emergency control; pliable enablers; steered activities; operation sphere influence; and creating a novel business model. This is expected to generate a medium-term stabilizing effect in the lodging industry even after health crisis outbreaks, and hence, opens the door for a sustained lodging sector recovery. How to make the change dimensions work? This can be accomplished through the combined effect of two types of interventions. Firstly, the management enabling factors materialized by handling the emergency; handling pliability; and enhancing the hotel managers’ impact. Secondly, by utilizing innovative health enabling trends in applying service androids, adopting service hygienic services, and adopting health-improved standing. Therefore, the input factors of management and health enablers; transformation via the change actions; output of improved managers’ abilities and hotel hygienic factors; outcome implication of medium-stabilizing effects; and impact effect of sustained lodging sector recovery, all form a chain of input, transform (actions), output, outcome, and impact result-chain reading of the framework.

#### The study practicable inferences

This study has important implications for hotels that are struggling with the current emergency. Those who seek to manage the emergency should get involved with those who practiced it. Corresponding to the situation parameters and changes caused by the “*Alpha”; “Beta”; “Gamma”; “Delta”; and “Omicron”* Corona Variants, the business is changing and evolving with luxury lodging services. In the short term, this evolution will be costly to the economy, as part of the jobs be replaced by new technologies.

Heightened market competition, together with any possible global economic downturn, may put those hotels that are merely prepared for emergencies on equal ground with others, and thus result in the survival of those hotel chains whose management and human resources are strongly sound. In this way, we’ll see new competitors rise to prominence, while others will fall by the wayside.

On a practical level, recovery strategic plans and processes defined globally by multinational and hotel chains’ headquarters or regional offices should be visible and well addressed. Informing the management team about all relevant information before initiating emergency management should be on the manager’s to-do list. Managers should be prepared and ready to make decisions, as well as to cope with the situation in terms of their ability to handle stress. To better prepare managers for a potential emergency relevant training should be provided.

This study discovered that luxury hotel managers who have dealt with past crises showed better emergency promptness competence in terms of clear thinking, rapid information flow, and the ability to see into the future. Despite everything, managers were always ready and willing to confront crises. Small hotels and motels that are unaware of the significance of emergency management generally do not have staff or departments devoted to emergency management. They may look at the administrative structures and procedures of other hotels to learn how to deal with an emergency.

To meet new market requirements, the lodging sector need to boost diversification in products and services. In most cases, a hotel’s opening or relaunch necessitates changes in multiple departments, especially in those that engage most frequently with guests.

The widespread use of complex technology will grow, leading to in a modern age in which luxury hotel amenities will increasingly be handled using devices that are not contact-dependent.

## Conclusion

This research offers a view of the luxury lodging industry at a time when most countries had just begun loosening travel restrictions, giving Egypt snapshot of the market. The study considered the effect that emergency management and pliability strategies have on administrative consequences in lodging industry. Additionally, given the complex nature of the Corona Variants attacks and their impact on the flow and nature of operations, the hotels’ planning process must be pliable and adaptable.

The study contributes to knowledge since it demonstrates the practical application of the broad inductive method of qualitative research. To achieve this goal, we combine the findings of our exploratory qualitative study with the interpretation of management enabling variables and health facilitating trends in the lodging sector. This is done in the context of a Framework for Health Crisis Management in the lodging sector to make the managers’ responses more generic. Value is also added by the health crisis management framework. By providing the framework for health crisis management, the study recommends an adjustment that can be indorsed via a result-chain repercussion. This is where the framework for sustained lodging sector recovery presented as the study’s main implication amalgamates a combination of input factors of management and health enablers; transformation via the change actions; output of containing the effect of the pandemic through enhanced managers’ capabilities and hotel health readiness; and an outcome implication of medium-stabilizing effects that leads to an impact effect of sustained lodging sector recovery. This configures an input; transformation (actions); output; outcome; and impact, resulting in a result-chain scrutiny.

## Data Availability

The authors declare they have full access to all study data, take full responsibility for the accuracy of the data analysis, and have authority over manuscript preparation and decisions to submit the manuscript for publication.

## Footnotes

1 “*On 26 November 2021, WHO designated the variant B.1.1.529 a variant of concern, named Omicron*” (“Update on Omicron”, 2022).
